# A Novel Strategy Based on Zn(II) Porphyrins and Silver Nanoparticles to Photoinactivate *Candida albicans*

**DOI:** 10.2147/IJN.S404422

**Published:** 2023-06-07

**Authors:** Bruno L Raposo, Sueden O Souza, Gleyciane S Santana, Max T A Lima, José F Sarmento-Neto, Júlio S Reboucas, Goreti Pereira, Beate S Santos, Paulo E Cabral Filho, Martha S Ribeiro, Adriana Fontes

**Affiliations:** 1 Departamento de Biofísica e Radiobiologia, Universidade Federal de Pernambuco, Recife, PE, Brazil; 2 Departamento de Química Fundamental, Universidade Federal de Pernambuco, Recife, PE, Brazil; 3 Departamento de Química, Universidade Federal da Paraíba, João Pessoa, PB, Brazil; 4 Departamento de Química & CESAM, Universidade de Aveiro, Aveiro, Portugal; 5 Departamento de Ciências Farmacêuticas, Universidade Federal de Pernambuco, Recife, PE, Brazil; 6 Centro de Lasers e Aplicações, Instituto de Pesquisas Energéticas e Nucleares (IPEN-CNEN/SP), São Paulo, SP, Brazil

**Keywords:** photodynamic inactivation, fungi, plasmon, photosensitizer

## Abstract

**Background:**

Photodynamic inactivation (PDI) is an attractive alternative to treat *Candida albicans* infections, especially considering the spread of resistant strains. The combination of the photophysical advantages of Zn(II) porphyrins (ZnPs) and the plasmonic effect of silver nanoparticles (AgNPs) has the potential to further improve PDI. Here, we propose the novel association of polyvinylpyrrolidone (PVP) coated AgNPs with the cationic ZnPs Zn(II) *meso*-tetrakis(*N*-ethylpyridinium-2-yl)porphyrin or Zn(II) *meso*-tetrakis(*N*-n-hexylpyridinium-2-yl)porphyrin to photoinactivate *C. albicans*.

**Methods:**

AgNPs stabilized with PVP were chosen to allow for (i) overlap between the NP extinction and absorption spectra of ZnPs and (ii) favor AgNPs-ZnPs interaction; prerequisites for exploring the plasmonic effect. Optical and zeta potential (ζ) characterizations were performed, and reactive oxygen species (ROS) generation was also evaluated. Yeasts were incubated with individual ZnPs or their respective AgNPs-ZnPs systems, at various ZnP concentrations and two proportions of AgNPs, then irradiated with a blue LED. Interactions between yeasts and the systems (ZnP alone or AgNPs-ZnPs) were evaluated by fluorescence microscopy.

**Results:**

Subtle spectroscopic changes were observed for ZnPs after association with AgNPs, and the ζ analyses confirmed AgNPs-ZnPs interaction. PDI using ZnP-hexyl (0.8 µM) and ZnP-ethyl (5.0 µM) promoted a 3 and 2 log_10_ reduction of yeasts, respectively. On the other hand, AgNPs-ZnP-hexyl (0.2 µM) and AgNPs-ZnP-ethyl (0.6 µM) systems led to complete fungal eradication under the same PDI parameters and lower porphyrin concentrations. Increased ROS levels and enhanced interaction of yeasts with AgNPs-ZnPs were observed, when compared with ZnPs alone.

**Conclusion:**

We applied a facile synthesis of AgNPs which boosted ZnP efficiency. We hypothesize that the plasmonic effect combined with the greater interaction between cells and AgNPs-ZnPs systems resulted in an efficient and improved fungal inactivation. This study provides insight into the application of AgNPs in PDI and helps diversify our antifungal arsenal, encouraging further developments toward inactivation of resistant *Candida* spp.

## Introduction

Photodynamic inactivation (PDI) has been an attractive approach to manage topical microbial infections. In PDI, a photosensitizer (PS) is excited by light at a wavelength overlapping with its absorbance, in the presence of molecular oxygen, resulting in the generation of reactive oxygen species (ROS) that leads to cellular killing.^1^

In particular, the tetracationic Zn(II) *meso*-tetrakis(*N*-ethylpyridinium-2-yl)porphyrin (ZnTE-2-PyP^4+^; ZnP-ethyl) and Zn(II) *meso*-tetrakis(*N*-n-hexylpyridinium-2-yl)porphyrin (ZnTnHex-2-PyP^4+^; ZnP-hexyl) were explored by us to photoinactivate *Leishmania* spp. and *Candida albicans*, with no noteworthy toxicity to mammalian cells.^1–4^ The promising results obtained were associated with the incorporation of cationic *meso-N*-alkylpyridinium substituents and the Zn(II) chelation in the porphyrin macrocycle, which (i) facilitate the PS interaction with the negatively charged microbial surface and (ii) increase the triplet lifetime, the ROS production, and the PS chemical stability.^1^,^5–7^ Notwithstanding the similarities of these ZnPs, ZnP-ethyl differs from ZnP-hexyl in its shorter aliphatic chain, with greater hydrophilicity, and usually requires higher molar concentrations for PDI applications.^2^,^3^

In addition to the design of PSs with improved cellular interaction and photophysical properties, innovative approaches employing nanotechnology have also been explored to enhance PDI efficiency.^8–11^ On this matter, silver nanoparticles (AgNPs) have been showing antimicrobial effect and ability to increase microbial photoinactivation when associated with PSs.^10^,^12–14^ Light can induce a collective oscillation of electrons of the conduction band of metallic NPs, known as the localized surface plasmon resonance (LSPR) effect.^9^ LSPR can amplify the electromagnetic field near the nanoparticles, boosting PS excitation and, consequently, ROS production.^15^ Nevertheless, more studies are needed to fully understand the role of AgNPs in PDI. Furthermore, few studies have evaluated the potential of AgNPs for PDI, especially in fungi.^10^,^15^

Therefore, herein, we evaluated the novel association of ZnPs (ZnP-ethyl or ZnP-hexyl) and AgNPs stabilized by polyvinylpyrrolidone (PVP) to photoinactive *C. albicans* yeasts. PVP-coated AgNPs were chosen to satisfy the necessary conditions to explore the LSRP in PDI. These include the overlap between the NP plasmon band and the PS absorption spectrum; and an NP-PS physical distance shorter than 10 nm.^16^ PVP is an anionic polymer which has been efficiently used for interaction with positively charged molecules.^17^

The fungal resistance has been a concern of growing relevance, although much less studied than bacterial resistance.^18^ *C. albicans* appears as the main cause of candidiasis and is among the most frequent mucocutaneous pathogens.^19^ This fungus can cause opportunistic infections, especially in susceptible persons, such as those undergoing immunosuppressing or antibiotic therapies, cancer treatment, diabetic patients, pregnant women, as well as those in the extremes of age.^20^,^21^ Recently, the World Health Organization (WHO) proposed a priority list for fungal pathogens with the aim to strengthen the fight against fungal infections and antifungal resistance.^22^ *C. albicans* is listed as the main fungus in the critical group due to its high infection rates and potential to develop resistance. Due to its importance for public health, we chose *C. albicans* as a biological model to investigate the PDI efficiency of our AgNPs-ZnPs systems.

In this way, we acknowledge the importance of broadening the tools available to combat fungal infections and believe that the contribution of AgNPs (and LSPR) to potentiate PDI mediated by ZnPs can significantly diversify the antifungal arsenal. Therefore, here, we applied PVP-coated AgNPs obtained by a simple synthetic route and demonstrated their ability to boost the PDI efficiency of two distinct ZnPs. To the best of our knowledge, this is the first report applying AgNPs and ZnPs for this purpose. We hope that the present study can contribute toward the control of fungal infections and to a better understanding of the role of AgNPs in PDI.

## Materials and Methods

### Synthesis and Characterization of AgNPs

The synthesis of AgNPs was carried out following the methodology described by Amirjani et al^23^ with adaptations. Briefly, AgNPs were produced by mixing trisodium citrate – TSC (1.5 mL, 30 mM, Dinâmica) and silver nitrate (AgNO_3_, 25.0 mL, 0.1 mM, Sigma-Aldrich) in an aqueous medium, at room temperature (~ 25 °C), under vigorous stirring. After 5 min, a solution of PVP (1.5 mL, 0.7 mM, Sigma-Aldrich, MW 29,000) was added, and then the reaction mixture was completed by adding a sodium borohydride solution (NaBH_4_, 0.15 mL, 100 mM, Sigma-Aldrich) previously refrigerated at 4 °C, changing the suspension from colorless to translucent yellow.

**Figure 1:**
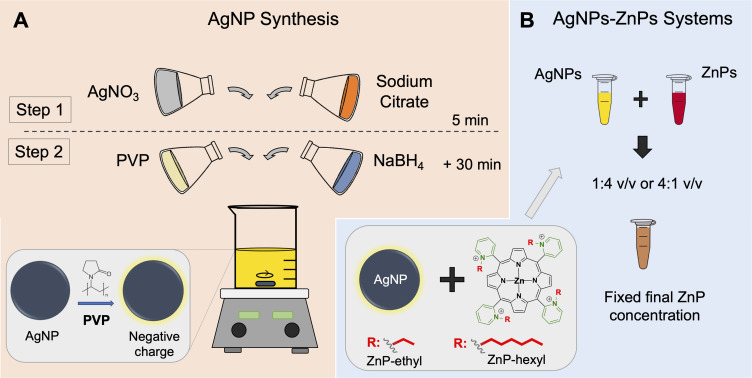
(**A**) Schematic representation of the synthesis of silver nanoparticles (AgNPs) coated with polyvinylpyrrolidone (PVP) and (**B**) the preparation of the AgNPs-ZnPs systems. ZnP = Zn(II) porphyrin.

Finally, the reaction mixture was kept under continuous stirring for 30 min to yield the AgNPs suspension (Figure 1A). The plasmon band of AgNPs was monitored by UV–Vis spectroscopy (Model 1800, Shimadzu) from 300 to 800 nm. Transmission electron microscopy (TEM) images were also acquired to analyze the morphology and size of the AgNPs (FEI Tecnai Spirit Bio-Twin, 120 kV). For this, the suspension of AgNPs was dripped onto a copper grid (01895, Ted Pella, Inc.) and allowed to dry before the analysis. The ImageJ software was used to measure the mean NP size. Inductively coupled plasma optical emission spectrometry (ICP-OES) (iCAP 6000 Series, Thermo Scientific) was used to quantify the Ag concentration in the NP suspension. After this, the concentration of AgNPs was estimated based on the mean size measured by TEM and the Ag quantification obtained from ICP-OES.

### Preparation of AgNPs-ZnPs Systems

The Zn(II) *meso*-tetrakis(*N*-n-hexylpyridinium-2-yl)porphyrin (ZnTnHex-2-PyP^4+^, ZnP-hexyl) and Zn(II) *meso*-tetrakis(*N*-ethylpyridinium-2-yl)porphyrin (ZnTE-2-PyP^4+^, ZnP-ethyl) were synthesized by alkylation of the precursor Zn(II) *meso*-tetrakis(2-pyridyl)porphyrin as described by Souza et al^1^ and characterized as previously reported.^1^,^2^,^24^,^25^ The concentrations of the aqueous ZnP solutions used throughout this work were determined spectrophotometrically using the published molar extinction coefficient for the Soret bands of ZnP-ethyl (ε_425.5 nm_ = 288,403 cm^−1^ M^−1^)^26^ and ZnP-hexyl (ε_427.0 nm_ = 436,516 cm^−1^ M^−1^).^24^

The suspension of AgNPs was filtered by centrifugation for 10 min at 2495 ×*g* (Universal Centrifuge 320 R, Hettich Zentrifugen) using concentrator tubes (10 kDa, Vivaspin^®^). The AgNPs were then resuspended in ultrapure water to half of the initial volume of the suspension. The resulting stock suspension of AgNPs contained *ca*. 0.9×10^12^ NPs/mL (see Results and Discussion section). Solutions of ZnPs were prepared in ultrapure water to yield the desired final porphyrin concentration upon mixing with the suspension of AgNPs at 1:4 or 4:1 v/v ratios (NPs:ZnPs). The volumes of AgNPs and ZnPs samples were maintained constant regardless of the PS concentration used (Figure 1B). The AgNPs-ZnPs suspensions were kept under gentle shaking (GyroMini, Labnet) for 48 h before application in PDI.

### Characterization of the AgNPs-ZnPs Systems

ZnPs and AgNPs-ZnPs systems were characterized by UV–Vis (SPECTROstar Nano, BMG Labtech) and emission (LS55, PerkinElmer) spectroscopies to obtain the spectral profile of the ZnPs alone or associated with the AgNPs. Furthermore, the zeta potential (ζ) of AgNPs alone and AgNPs-ZnPs systems was also evaluated (Zetasizer NanoZS, Malvern Panalytical). For these characterizations, systems were prepared to reach the highest PS concentrations to be tested in PDI with NPs, *i.e.*, 3.0 µM for ZnP-ethyl and 1.6 µM for ZnP-hexyl (at 1:4 or 4:1 v/v ratio). The pH values of the systems were measured before the analyses and kept at approximately 7.0. The ζ measurements included at least four replicates per group.

ROS generation was assessed for individual ZnPs or AgNPs-ZnPs systems (1:4 v/v ratio), using the probe 2′,7′-dichlorofluorescin diacetate – DCFH-DA (Sigma-Aldrich). To allow for comparison, the same PS concentration (1.6 µM) was used for both ZnPs, with or without AgNPs. The systems (ZnP-hexyl, ZnP-ethyl, AgNPs-ZnP-hexyl or AgNPs-ZnP-ethyl) were added to a black polystyrene 96-well microplate (Optiplate HB – PerkinElmer). Before the irradiation, DCFH-DA probe, prepared according to the manufacturer’s instructions, was added to the selected wells to reach a final concentration of 10 µM. The samples were irradiated following PDI parameters described later in this methodology. Forty minutes after irradiation, the probe fluorescence was quantified using a microplate reader (Varioskan, Thermo Fisher) with F485 (excitation) and F530 (emission) filters. ROS measurements were performed in quadruplicate.

### *Candida albicans* Growth Conditions and Preparation for PDI

Prior to each experiment, *C. albicans* cells (ATCC 90028) were cultured in Sabouraud Dextrose Broth medium (SDB, Neogen) at 37 °C for 18 h. Then, the yeast suspension was centrifuged at 600 ×*g* for 1 min (MiniSpin, Eppendorf) and the cell pellet was resuspended in 1× phosphate-buffered saline (PBS) and diluted to achieve a concentration of 1×10^7^ colony forming units per milliliter (CFU/mL). The yeast concentration was confirmed by optical density (OD_540_) (MQX200 µQUANT microplate, BioTeck), which was previously standardized by cell counting in a Neubauer chamber.

### Photoinactivation of *Candida albicans*

Biological assays were performed in 96-well microplates containing 100 µL of *C. albicans* yeast suspension (1×10^7^ CFU/mL) and 100 µL of PBS or one of the prepared systems (ZnPs, AgNPs or AgNPs-ZnPs). The experimental groups included (1) Control: yeasts with no irradiation nor treatment; (2) Light: cells + irradiation; (3) Dark: yeasts with the systems (ZnPs or AgNPs-ZnPs) without irradiation; (4) AgNPs/L+: cells + nanoparticles + irradiation; and (5) PDI: yeasts with the systems (ZnPs or AgNPs-ZnPs) + irradiation. To investigate the effect of the association of AgNPs and ZnPs in PDI, we chose to keep the pre-irradiation incubation time (PIT) and irradiation parameters (IR) fixed; and two AgNPs:ZnPs proportions (systems 1:4 and 4:1 v/v, as previously explained) and various ZnP concentrations were evaluated. The maximum final concentrations of ZnPs tested in PDI assays with AgNPs were 0.8 µM for ZnP-hexyl and 1.5 µM for ZnP-ethyl (regardless of the AgNPs:ZnPs ratio). The PIT and IR conditions applied in this work were based on a previous study carried out by us.^3^ To simultaneously photoexcite the ZnPs and induce the LSPR phenomenon in AgNPs, a blue LED (410 ± 20 nm, LEDbox, BioLambda) was used, as this wavelength range overlaps with the absorption of ZnPs and the plasmon band of AgNPs. Yeast suspensions were pre-incubated with the ZnPs, AgNPs or AgNPs-ZnPs systems for 10 min, and then immediately irradiated for 3 min (irradiance of 24.1 mW/cm^2^ and a light dose of 4.3 J/cm^2^). For the control and dark groups, the total incubation time was 13 min.

As the systems containing AgNPs were diluted by half upon mixing with the yeast suspension, the whole biological medium (PS + yeast) containing AgNPs-ZnPs at 1:4 or 4:1 v/v had a total of *ca*. 0.2×10^12^ NPs/mL and 0.7×10^12^ NPs/mL, respectively. Likewise, the concentration of ZnPs in the AgNPs-ZnPs stock suspensions was twice as high as the one intended to be used in the biological assay to account for the 1:1 dilution upon mixing with the yeast suspension.

The PDI effect on *C. albicans* was assessed following the method proposed by Jett et al^27^ Briefly, serial dilutions of the samples were obtained in PBS, and 10 µL of each dilution was seeded onto Sabouraud Dextrose agar, and incubated for 24 h at 37 °C. The CFUs were then counted, adjusted by the dilution factor, and represented as logarithms (log_10_). At least three independent experiments were performed with three replicates per group.

### Microscopy Analyses

The interaction of the ZnPs or AgNPs-ZnPs systems with *C. albicans* cells was evaluated by confocal fluorescence microscopy. For this analysis, each system – ZnPs alone or AgNPs-ZnPs (1:4 v/v) – contained a final PS concentration of 5 µM. First, yeast suspensions in PBS (1×10^7^ CFU/mL) were allowed to adhere to the bottom of microscopy plates (CELLview, Greiner Bio-one) for 60 min at 37 °C. The plates were washed to remove non-adherent cells and incubated with the PS systems for the same time used in PDI. Then, the samples were washed twice with PBS to remove the excess of ZnPs or AgNPs-ZnPs systems, and analyzed under a confocal microscope (FV 1000, Olympus), using an excitation at 473 nm and a 63×/NA = 1.35 objective.

### Statistical Analyses

To analyze the data for normal distribution, the Shapiro–Wilk test was performed, and statistical differences between the experimental groups were evaluated using the Mann–Whitney test (GraphPad Prism 7). The significance level was set as 0.05.

## Results and Discussion

### Characterization of AgNPs and AgNPs-ZnPs

**Figure 2:**
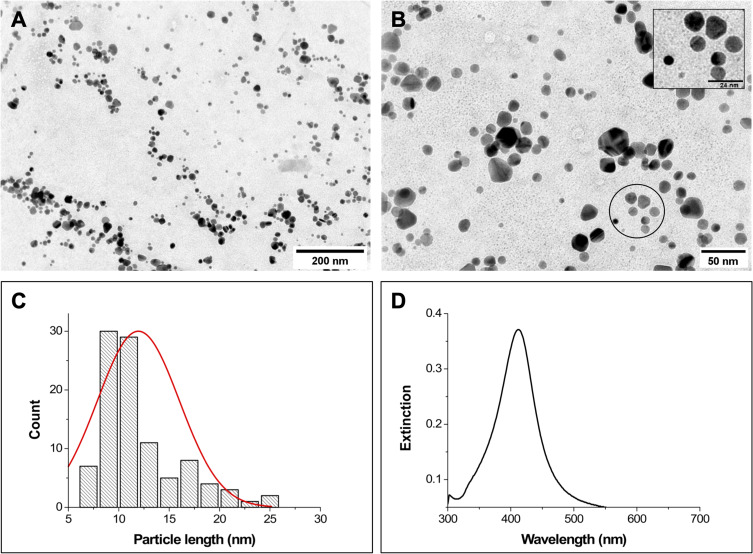
(**A**) and (**B**) Representative TEM micrographs of AgNPs, scale bars = 200 and 50 nm, respectively; inset scale bar = 24 nm. (**C**) Particle size distribution obtained from TEM images (total particles measured = 100). (**D**) Extinction spectrum of AgNPs.

Based on the available literature, it is expected that spherical AgNPs present an extinction band close to 400 nm.^28^,^29^ The AgNPs synthesized here presented a morphology predominantly spherical (Figures 2A and B) and a mean diameter of *ca*. 14 nm (Figure 2C). The AgNPs also presented a plasmon band at 413 nm (Figure 2D), which corroborates the spherical morphology observed in the TEM analysis. Using the mean size determined by TEM and the Ag quantification obtained by ICP-OES, a concentration of approximately 0.9×10^12^ AgNPs/mL was estimated. These morphological and spectral characteristics of the AgNPs synthesized in this study corroborate the data presented in the literature.^23^

**Figure 3:**
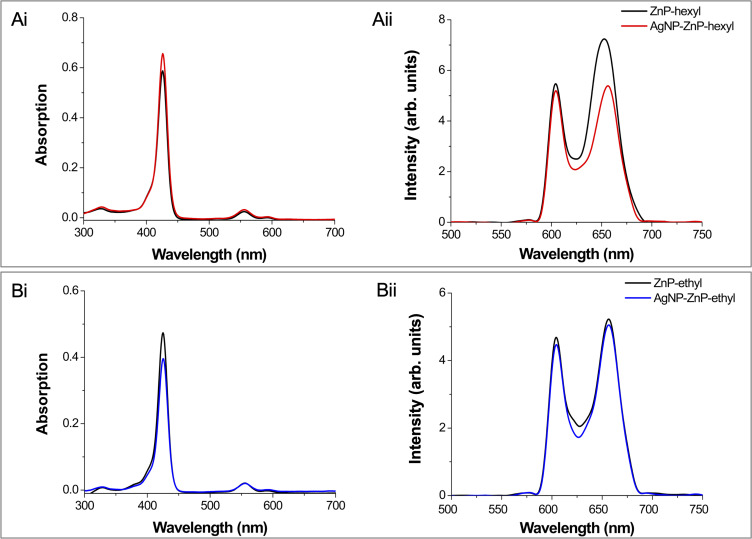
Absorption and emission spectra of ZnP-hexyl based systems (**Ai** and **Aii**, respectively) and ZnP-ethyl based systems (**Bi** and **Bii**, respectively). Data for each ZnP alone (black) and the systems AgNPs-ZnP-hexyl (red) and AgNPs-ZnP-ethyl (blue) were acquired in water using the following conditions, ZnP-hexyl at 1.6 µM, ZnP-ethyl at 3.0 µM, and AgNPs-ZnPs 1:4 v/v. Emission spectra were acquired at λ_exc_ = 426 nm.

Figure 3 shows the optical characterization of ZnPs and AgNPs-ZnPs systems (1:4 v/v). The absorption spectra of both ZnPs showed the three bands characteristic of Zn(II) porphyrins: the Soret band and two less intense bands, α and β.^1^,^24^,^25^,^30^ Moreover, the emission spectra of both ZnPs showed two intense bands in the red spectral region, similar to those already described elsewhere.^2^,^30^ The association of ZnPs with AgNPs did not cause considerable changes in the absorption spectra of the PSs. After interaction with AgNPs, ZnP-ethyl displayed a similar emission spectral profile, with a discreet decrease in fluorescence intensity (Figure 3Bii). This reduction was, however, higher for the second emission peak of ZnP-hexyl (Figure 3Aii). Similar spectral changes were observed for both ZnPs with a 4-fold increase of AgNPs amount in the mixture (4:1 v/v; data not shown).

The LSPR phenomenon of metallic NPs has been one of their most explored properties for biomedical applications.^31^ The LSPR phenomenon may modulate the optical profile of fluorescent compounds, such as a PS, when they are placed close to the metallic NPs. This interaction may result in the decrease or enhancement of the fluorescence intensity, for example. In this way, spectral changes may suggest that there was an interaction between ZnPs and AgNPs.^9^,^32–34^

**Figure 4:**
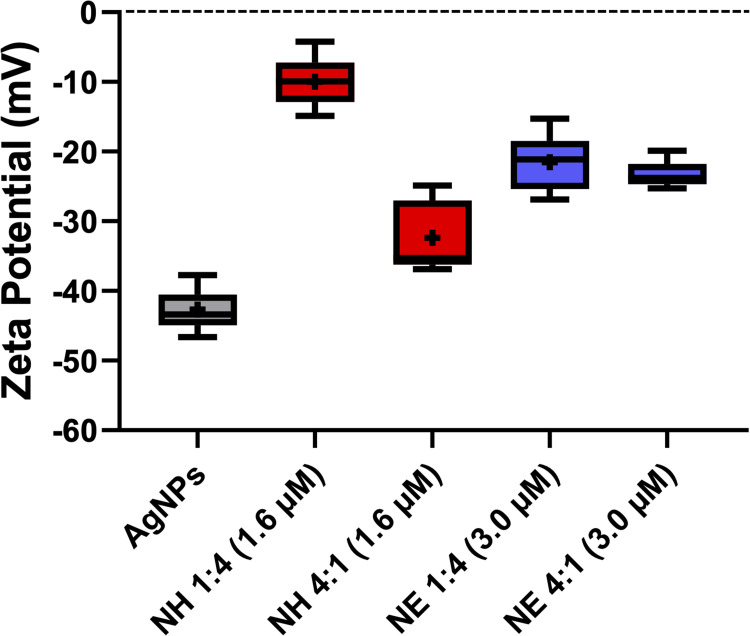
Box plots of the zeta potential values obtained for AgNPs alone and the AgNPs-ZnPs systems. All groups showed statistically significant differences compared to AgNPs (p < 0.05). The final ZnP concentrations are shown in parentheses. NH: AgNPs-ZnP-hexyl; NE: AgNPs-ZnP-ethyl.

Another characterization included the measurements of ζ, which can be used to monitor electrical changes on the surface of NPs and infer on their interaction with different compounds, such as PSs.^35^ Figure 4 shows the ζ results for AgNPs and AgNPs-ZnPs systems. When compared to AgNPs alone (ζ = –43.4 mV), AgNPs-ZnPs systems at a 4:1 v/v mixture showed less negative net charges; the ζ values were –35.3 and –24.5 mV when ZnP-hexyl and ZnP-ethyl were associated with NPs, respectively (under these conditions, there were *ca*. 1.3×10^3^ and 2.5×10^3^ ZnP per AgNP). In the mixtures in which cationic ZnPs were proportionally more available (1:4 v/v), a further reduction of ζ values (in modulus) was observed; the measurements yielded ζ = –9.6 mV for the ZnP-hexyl system (containing *ca*. 5.3×10^3^ molecules of ZnP per AgNP) and ζ = –22.8 mV for the ZnP-ethyl system (containing *ca*. 10.0×10^3^ ZnP per AgNP). As described in the Material and Methods section, the concentrations of ZnP-hexyl (1.6 µM) and ZnP-ethyl (3.0 µM) in ζ measurements were chosen to correspond to the highest PSs concentrations associated with AgNPs for PDI (before dilution by half with the yeast suspension).

The polymer PVP confers negative charges to the NP surface, allowing for their interaction with cationic compounds.^17^,^36^ Therefore, the ζ changes observed for the systems containing ZnPs suggest that an interaction between the cationic porphyrins and AgNPs is taking place in all conditions tested. A similar behavior was observed by Rigotto-Caruso et al,^10^ who reported a reduction of the ζ absolute value of AgNPs due to the presence of cationic phenothiazines. Likewise, Rodrigues et al^9^ also observed a reduction in the ζ modulus when Ag nanoprisms were associated with the cationic dye methylene blue.

Moreover, the spectroscopic and ζ results suggest that ZnP-hexyl seems to have greater interaction with AgNPs. In addition to the electrostatic attraction, we believe this behavior may be explained by a further contribution of intermolecular interactions, such as London dispersion forces, between the larger alkyl chain of ZnP-hexyl and the organic chains of the PVP polymer that coats AgNPs.^37^,^38^ Furthermore, differences observed in ζ, related to the porphyrin could also be due to the longer aliphatic chains of ZnP-hexyl, which afford a more hydrophobic compound, reducing the NP negative surface charge and favoring a steric stabilization.^39^,^40^

### Photoinactivation of *Candida albicans*

**Figure 5:**
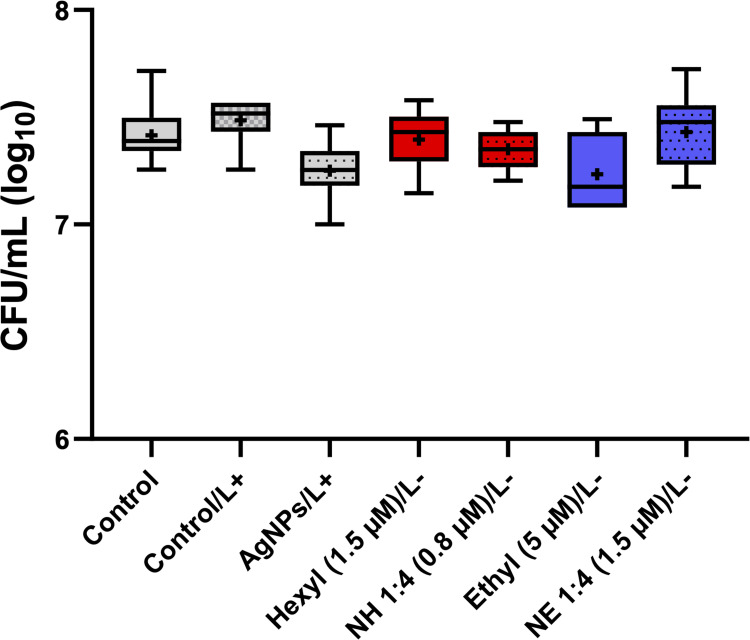
Box plots of *C. albicans* yeast cells after incubation with PSs (ZnP-hexyl or ZnP-ethyl) associated or not with AgNPs, in the dark. The pre-irradiation incubation time was 10 min. The plot also expresses the proliferation of yeasts exposed to (i) no irradiation nor treatment – Control; (ii) light alone (4.3 J/cm^2^) – Control/L+; and (iii) AgNPs plus irradiation – AgNPs/L+. The final concentrations of ZnPs applied are shown in parentheses. L+: with irradiation (light dose 4.3 J/cm^2^); L-: without irradiation. Hexyl: ZnP-hexyl; Ethyl: ZnP-ethyl; NH: AgNPs-ZnP-hexyl; NE: AgNPs-ZnP-ethyl. At least three independent experiments were performed in triplicate.

The effects of AgNPs against microorganisms, such as bacteria, have been shown to be dependent on certain NP parameters such as charge, concentration, size, surface area, and morphology.^41^ Furthermore, the interaction of NPs with the microbial cell surface appears to be paramount for the antimicrobial effect to occur.^41–44^ In our study, the results showed that *C. albicans* cells were not noteworthily affected by AgNPs after irradiation with a light dose of 4.3 J/cm^2^ (Figure 5), possibly due to the short interaction time (total 13 min) of the yeasts with these nanostructures.

Our results corroborated literature reports that observed minimal antimicrobial effect of AgNPs when short interaction times were used. Astuti et al^45^ evaluated the inactivation of *C. albicans* yeasts assisted by AgNPs under blue laser irradiation (450 nm, IR time of 1.25 min, and 29.25 J/cm^2^). Their results showed *ca*. 1 log_10_ inactivation of yeasts. Similarly, Rigotto-Caruso et al^10^ found that biosynthesized spherical AgNPs did not show great effects in inhibiting the growth of *C. albicans* (ATCC 64548), with or without irradiation by a red LED (635 nm, PIT of 30 min, IR time of 16.41 min, and 15 J/cm^2^).

Our data showed that light alone (4.3 J/cm^2^) and systems without light irradiation caused no noteworthy effect on *C. albicans* yeasts (Figure 5). In a previous study, it was already verified that ZnP-hexyl alone, at the same concentration tested here (1.5 µM), did not present cytotoxicity for *C. albicans* (ATCC 90028) in the dark.^3^ Similarly to our results, Viana et al^2^ did not observe an antimicrobial effect on *C. albicans* (ATCC 10231) when a higher concentration of ZnP-ethyl (10 µM) was applied in the absence of irradiation.

**Figure 6:**
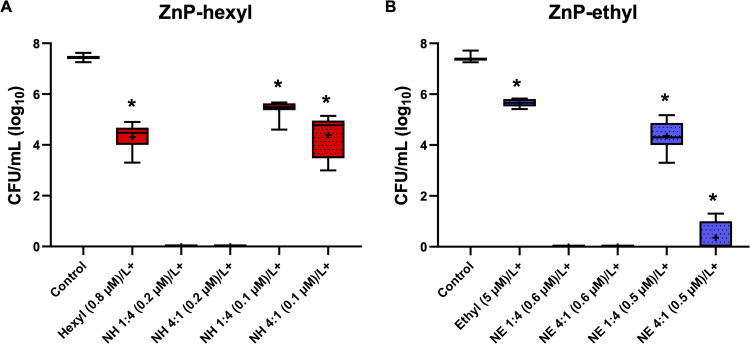
Box plots of photoinactivation of *C. albicans* yeasts. The photoefficiency was evaluated with individual ZnPs (**A**) ZnP-hexyl at 0.8 µM and (**B**) ZnP-ethyl at 5.0 µM or in association with AgNPs (1:4 and 4:1 v/v ratio) at different PS concentrations. ZnP concentrations are given in parenthesis. The pre-irradiation incubation time was 10 min and the light dose was 4.3 J/cm^2^. Hexyl: ZnP-hexyl; Ethyl: ZnP-ethyl; NH: AgNPs-ZnP-hexyl; NE: AgNPs-ZnP-ethyl. L+: with irradiation. At least three independent experiments were performed in triplicate; *p < 0.05 in relation to the control.

On the other hand, a reduction of ~3 log_10_ was observed (Figure 6A) when cells were irradiated in the presence of ZnP-hexyl (0.8 µM). This result was similar to that reported by Souza et al^3^ when ZnP-hexyl was evaluated as PS with the same yeast strain and under similar PDI conditions. A more discrete reduction (2 log_10_) was achieved with ZnP-ethyl under irradiation, despite using a higher PS concentration (5 µM) (Figure 6B). Thus, although both ZnPs promoted an antimicrobial photodynamic effect on *C. albicans*, ZnP-hexyl showed a higher efficiency. Similar results of PDI using ZnP-ethyl were noted by Viana et al^2^ who verified a reduction of 3 log_10_ in *C. albicans* (ATCC 10231) using a higher concentration of ZnP-ethyl (10 µM) and irradiation time (9 min; 150 mW/cm^2^, blue LED). The difference observed between the PDI mediated by ZnP-hexyl and ZnP-ethyl can be related to their structural characteristics. The increase in the length of the alkyl aliphatic side chain of the porphyrins (*i.e.*, n-hexyl versus ethyl) confers a more amphiphilic character to these PSs, leading to greater cell interaction with ZnP-hexyl,^24^ which may contribute to the better efficiency observed here for PDI mediated by ZnP-hexyl. ZnP-ethyl, on the other hand, has a more hydrophilic character due to its shorter side chain.^4^ The structural differences presented by these ZnPs and their efficiency in photoinactivation have also been described in other microorganisms, such as *Leishmania* spp.^1^

For systems containing NPs, different concentrations of ZnPs associated with AgNPs at different proportions were evaluated for PDI (Figure 6). As previously described, AgNPs-ZnPs suspensions were prepared from stock samples of AgNPs and ZnPs, which ultimately yielded systems with lower proportion of AgNPs (1:4 v/v) or 4-fold more AgNPs (4:1 v/v). We found that when AgNPs were associated with ZnPs, regardless of the proportion, a considerable improvement in the PDI effect was observed, using the same light parameters.

PDI mediated by AgNPs-ZnP-hexyl systems demonstrated an antifungal effect at very low PS concentrations. Under the applied conditions, these systems promoted *C. albicans* eradication at a PS concentration as low as 0.2 µM for both 1:4 and 4:1 v/v AgNPs:ZnP-hexyl. When a PS concentration of 0.1 µM was used with a lower proportion of AgNPs (*i.e.*, 1:4 v/v mixture), a 2 log_10_ CFU reduction was observed; whereas the 4-fold increase in AgNPs in this system (*i.e.*, 4:1 v/v mixture) promoted a higher CFU reduction of 3 log_10_ (Figure 6A).

Moreover, under the conditions tested, the AgNPs-ZnP-ethyl systems also proved efficient to completely photoinactivate *C. albicans* at PS concentrations as low as 0.6 µM (Figure 6B), with either lower or higher proportion of AgNPs. When using a lower concentration of ZnP-ethyl (0.5 µM) and lower AgNPs amount (*i.e.*, AgNPs-ZnP-ethyl 1:4 v/v), a reduction of approximately 3 log_10_ was observed. On the other hand, when the AgNPs:ZnPs proportion was increased 4-fold (4:1 v/v), the same ZnP-ethyl concentration of 0.5 µM promoted an expressive improvement in the yeast photoinactivation, with a reduction of approximately 6.5 log_10_.

According to the results summarized in Figure 6, when a greater proportion of AgNPs *(i.e.*, AgNPs-ZnP mixtures at 4:1 v/v) was employed, an improvement in PDI was verified when compared to the corresponding 1:4 v/v ratio (p < 0.05) for smaller ZnP concentrations. Furthermore, our data suggested that AgNPs seem to have a higher impact on PDI mediated by ZnP-ethyl than on that by ZnP-hexyl. This is evidenced when comparing the magnitude of change in PS concentration between ZnP alone and those groups associated with AgNPs: (i) ZnP-ethyl eradicated yeasts at a concentration ~8× lower (ZnP-ethyl = 0.6 µM) than the one applied without AgNPs (ZnP-ethyl = 5 µM); (ii) at the same proportions of AgNPs used in the ZnP-ethyl systems, the corresponding AgNPs-ZnP-hexyl required PS concentrations ~4× lower (ZnP-hexyl = 0.2 µM) than that without NPs (ZnP-hexyl = 0.8 µM). Thus, our results clearly showed that association with AgNPs made it possible to reduce the effective concentrations of both ZnPs and still preserve (or enhance) PDI efficiency. It is also worth noting that, although ZnP-hexyl alone is already a better PS than individual ZnP-ethyl, the use of AgNPs may represent a viable strategy to considerably boost the efficiency of less effective PSs, such as, in the present case, ZnP-ethyl. Thus, association with AgNPs allowed the photoeradication of *C. albicans* at submicromolar concentrations of ZnPs.

Few studies in the literature sought to evaluate the action of PSs associated with metallic NPs, such as AgNPs, on fungal inactivation.^46–49^ Maliszewska et al^50^ evaluated the interaction of bimetallic NPs of Ag and Au (produced by *Trichoderma koningii*) linked to di-[(Al(OH)PC(SO_3_Na)_2_)–AlPcS_2_] or trisulfonated [(Al(OH)PC(SO_3_Na)_3_)–AlPcS_3_] phthalocyanine (PC) for PDI of *C. albicans* (ATCC 10231) yeasts. The study used a laser at 650 nm (12.6 to 94.5 J/cm^2^) and PIT of 30 min. For PDI studies, only the concentration of 7 mg/L of PCs was used. The authors found that there were no great effects when NPs were applied alone. The best result was obtained for the combination with NPs-AlPcS_2_, which led to a complete fungal inactivation at a light dose of 63 J/cm^2^. The authors stated that the better effect verified for the NP system containing AlPcS_2_ was due to the PS structure, which allowed a better interaction with the cells. Mapukata et al^8^ evaluated the PDI mediated by AgNPs linked to PCs on *C. albicans* (ATCC 24433) yeasts. In their work, a neutral ZnPC 2,9(10),16(17),23(24)-tetrakis-(4’-(4’-6’-diaminopyrimidin-2’-ylthio)) phthalocyaninate Zn(II) (ZnPC-neutral) and two other positively charged ZnPCs (ZnPC-hexadeca and ZnPC-octa) were used. The PCs-conjugate concentration used was 5 μM. The highest efficiency was observed when NPs were associated with ZnPC-hexadeca, which led to a 6.8 log_10_ reduction (PIT 80 min; 3.6 kJ/cm^2^). The better photodynamic effect was attributed to the ROS production and the cationic character of ZnPC-hexadeca which may facilitate the interaction of the PS with microbial cells.

PDI protocols that can be effective in eradicating fungal communities using reduced PS concentrations and milder irradiation parameters have been evaluated in the literature also to minimize local phototoxicity to host tissues.^30^,^31^ Our study showed that the PDI protocol based on the association of ZnPs with AgNPs was effective in promoting the eradication of *C. albicans* yeasts using submicromolar concentrations of ZnPs, short PIT, and low light dose.

Not much is yet known about the mechanisms involved in PDI when PSs are associated with metallic NPs for antimicrobial inactivation. The LSPR effect can enhance the electromagnetic field near the metallic NPs, which may improve the excitation of PSs near the NP surface and, consequently, increase ROS production.^12^,^31^,^51–53^ DCFH-DA is a probe widely used to assess the increase or decrease in ROS production. Although this probe may have some limitations, it can be used to infer about differences in ROS production between oxidative components.^54–56^

**Figure 7:**
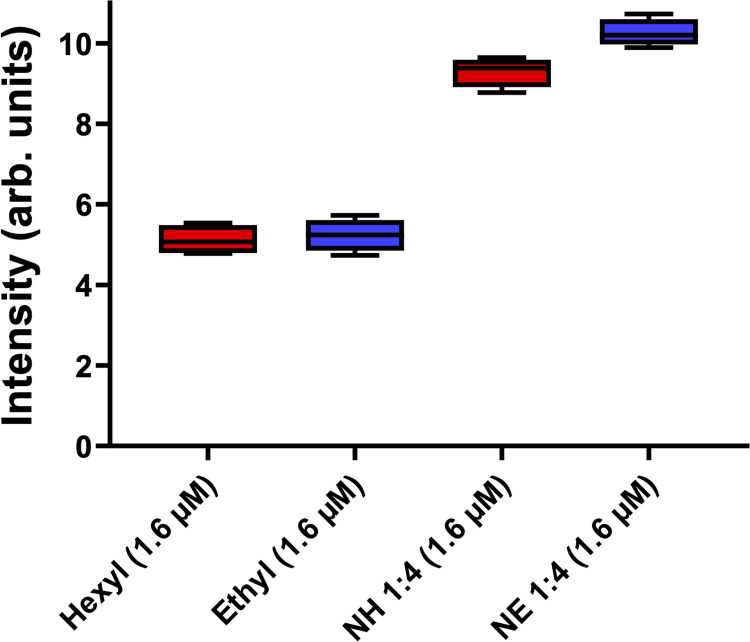
Box plots of the fluorescence intensity values obtained using the DCFH-DA probe for the ZnPs alone or AgNPs-ZnPs systems. Final ZnP concentrations are shown in parentheses. Hexyl: ZnP-Hexyl; Ethyl: ZnP-ethyl; NH: AgNPs-ZnP-hexyl; NE: AgNPs-ZnP-ethyl.

In our results using DCFH-DA (Figure 7), no differences were observed between ZnPs (hexyl or ethyl) with respect to ROS production. This suggests that the higher effectiveness of ZnP-hexyl in inactivating fungi may be related to its more lipophilic character, which favors greater cell interaction, causing lethal damage to essential organelles.^7^,^24^,^25^ Moreover, an increase in the ROS level was observed when ZnPs were associated with AgNPs (about 50% for both porphyrins). Thus, we hypothesize that this outcome may have a contribution to the greater performance observed for PDI of *C. albicans* when AgNPs were associated with ZnPs, but further studies are needed for definitive mechanistic conclusions.

### Microscopy Analyses

**Figure 8:**
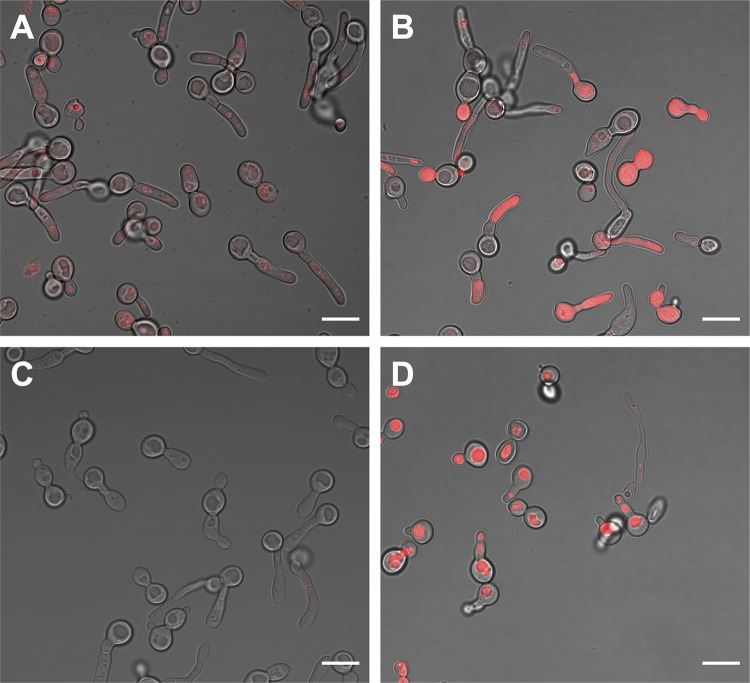
Representative confocal microscopy images of *C. albicans* yeast cells after incubation for 10 min with: (**A**) ZnP-hexyl; (**B**): AgNPs-ZnP-hexyl; (**C**): ZnP-ethyl; (**D**): AgNPs-ZnP-ethyl. AgNPs:ZnPs were applied at a ratio of 1:4 v/v. Scale bar: 10 µm.

We used the intrinsic fluorescence of the porphyrins to study and compare the cellular uptake/interaction of ZnPs vs AgNPs-ZnPs systems with *C. albicans* yeasts through fluorescence confocal microscopy. We found that using either ZnP-hexyl or ZnP-ethyl at the same concentration of 5 µM, only ZnP-hexyl was able to produce a detectable fluorescent labeling in the yeast cells (Figure 8A); no noteworthy labeling was observed upon incubation with ZnP-ethyl (Figure 8C). These differences may be related to the structural characteristics of these porphyrins, as ZnP-hexyl is more lipophilic, which favors its interaction with cells, in contrast with the more hydrophilic ZnP-ethyl.^24^ Similar results were noted by Souza et al^1^ in promastigotes of *L. amazonensis*, as they observed an intense labeling by ZnP-hexyl but not by ZnP-ethyl in the parasites.

When AgNPs-ZnP-hexyl was incubated with *C. albicans* yeasts, it was possible to observe an even more intense labeling (Figure 8B). Moreover, effective cell labeling was also observed after incubation with the AgNPs-ZnP-ethyl system (Figure 8D), as opposed to ZnP-ethyl alone (Figure 8C).

The fluorescent labeling found for the AgNPs-ZnP-ethyl system was unexpected, and we hypothesized that this result may be related to a possible mechanism of ZnP-ethyl delivery by AgNPs. The concentration of the PS on the NP surface may facilitate and direct its interaction with the cells. This also seems to agree with the AgNPs-ZnP-hexyl systems, which produced brighter fluorescent labeling than ZnP-hexyl alone.

Taken together, we believe that the PDI improvement observed by us after applying the AgNPs-ZnPs systems may be a combination of (i) the plasmonic effect, which led to a greater ROS production, plus (ii) an enhanced PS–cell interaction and PS intracellular availability provided by the nanostructures, which also collaborated to increase damage to subcellular structures.

## Conclusion

The problems associated with infections caused by *C. albicans* and the worrying emergence of resistant strains have encouraged the search for novel therapeutic methods that can be efficient and do not induce resistance. The present study reported a promising and improved photoinactivation of *C. albicans* yeasts using PDI mediated by novel systems comprised ZnPs (ZnP-hexyl or ZnP-ethyl) and AgNPs, applying submicromolar PS concentrations and mild irradiation parameters. We also believe that our study provided insights about the role of metallic NPs in PDI, encouraging further investigations using the proposed photonic strategy for other *Candida* species and resistant isolates, in planktonic forms and biofilms.

## Data Sharing Statement

The data generated during this study are included in this article and in its supporting information openly available in Zenodo at https://doi.org/10.5281/zenodo.7523409.
